# Targeted Biodegradable Near-Infrared Fluorescent Nanoparticles for Colorectal Cancer Imaging

**DOI:** 10.1021/acsabm.4c00072

**Published:** 2024-04-04

**Authors:** Seock-Jin Chung, Kay Hadrick, Md Nafiujjaman, Ehsanul Hoque Apu, Meghan L. Hill, Md Nurunnabi, Christopher H. Contag, Taeho Kim

**Affiliations:** † Department of Biomedical Engineering, Institute for Quantitative Health Science and Engineering, Michigan State University, East Lansing, Michigan 48824, United States; ‡ Department of Pharmaceutical Sciences, School of Pharmacy, University of Texas at El Paso, El Paso, Texas 79902, United States; § Department of Microbiology, Genetics and Immunology, Michigan State University, East Lansing, Michigan 48824, United States

**Keywords:** colorectal cancer, near-infrared, silica nanoparticles, biodegradable, carcinoembryonic antigen

## Abstract

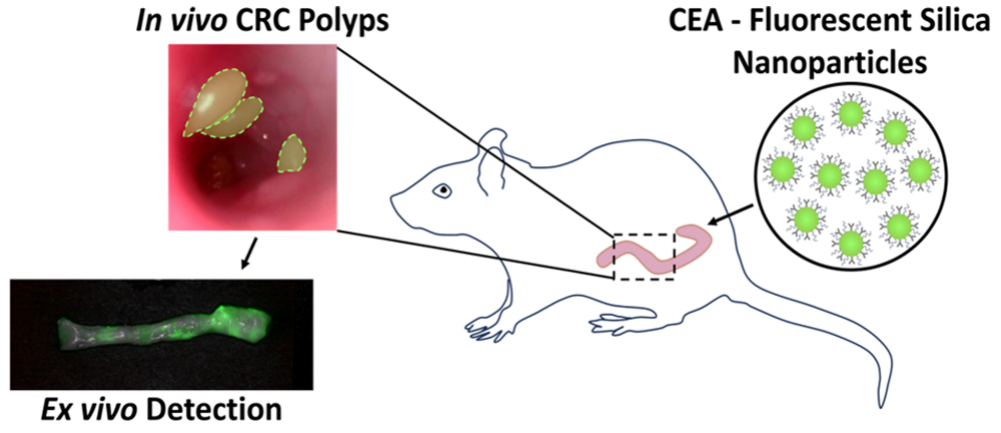

Colorectal cancer (CRC) is the third leading cause of cancer death in the U.S., and early detection and diagnosis are essential for effective treatment. Current methods are inadequate for rapid detection of early disease, revealing flat lesions, and delineating tumor margins with accuracy and molecular specificity. Fluorescence endoscopy can generate wide field-of-view images enabling detection of CRC lesions and margins; increased signal intensity and improved signal-to-noise ratios can increase both speed and sensitivity of cancer detection. For this purpose, we developed targeted near-infrared (NIR) fluorescent silica nanoparticles (FSNs). We tuned their size to 50–200 nm and conjugated their surface with an antibody to carcinoembryonic antigen (CEA) to prepare CEA-FSNs. The physicochemical properties and biodegradable profiles of CEA-FSN were characterized, and molecular targeting was verified in culture using HT29 (CEA positive) and HCT116 (CEA negative) cells. CEA-FSNs bound to the HT29 cells to a greater extent than to the HCT116 cells, and smaller CEA-FSNs were internalized into HT29 cells more efficiently than larger CEA-FSNs. After intravenous administration of CEA-FSNs, a significantly greater signal was observed from the CEA-positive HT29 than the CEA-negative HCT116 tumors in xenografted mice. In F344-PIRC rats, polyps in the intestine were detected by white-light endoscopy, and NIR fluorescent signals were found in the excised intestinal tissue after topical application of CEA-FSNs. Immunofluorescence imaging of excised tissue sections demonstrated that the particle signals coregistered with signals for both CRC and CEA. These results indicate that CEA-FSNs have potential as a molecular imaging marker for early diagnosis of CRC.

## 1 Introduction

Despite screening recommendations, colorectal cancer (CRC) is increasing in prevalence with alarming increases in younger people and is, at present, the third leading cause of cancer death in the United States.^1^ Over the last three decades, the death rate from CRC has dropped largely because colorectal polyps can be detected, and removed, during colonoscopy, which prevents progression to cancer.^2−4^ When CRC is found at an early stage, before it has become invasive, the 5-year relative survival rate is about 90%;^5,6^ when the cancer is detected after invasion of the colonic or rectal mucosa, survival rates drop to 14%.^7^ Early detection and diagnosis of colorectal cancer are essential for successful treatment; however, small and flat lesions can be missed, and molecular determinants cannot be assessed during colonoscopy.

Detection of CRC has currently improved with advances in computed tomography colonography,^8^ sigmoidoscopy,^9,10^ stool DNA test,^11^ fecal occult blood testing,^12^ and colonoscopy (the gold standard).^13^ Despite significant progress, CRC patients are often found in advanced stages, and additional tools are needed to improve the survival rate.^14^ Detection of lesions with white-light endoscopy depends on distinct anatomic anomalies, which are absent with serrated adenomas, which tend to be flat, or when lesions are small and indistinguishable from normal tissue.^15^ Dramatic anatomic changes occur late in disease stages whereas molecular markers can accompany malignant transformation, and therefore the collected data from endoscopic diagnosis would enable clinicians to both visualize and characterize lesions. Fluorescence endoscopy is a relatively new medical imaging technique that uses photosensitizing molecular probes and spectrally resolved illumination and detection to visualize topological abnormalities coregistered with molecular abnormalities. When guided by molecular screening tools with stool or blood, effective fluorescence endoscopy tools would greatly improve early detection as well as diagnosis and prognosis of CRC.

Fluorescence molecular imaging is noninvasive, relying on nonionizing radiation. Its attributes include rapidity, simplicity, cost-effectiveness, and sensitivity^16^ but is limited to signals that are relatively superficial given the limited penetration of light through mammalian tissue. Molecular probes used for fluorescence imaging include fluorescent dyes, proteins, or nanoparticles that can be used as labels to mark biological molecules such as nanobodies or antibodies.^17,18^ Fluorescent labeling enables the tagged biomolecules (signal) to be differentiated from nonfluorescent or less fluorescent materials or structures (noise) in the body.^19^ In previous studies, a biodegradable fluorescent nanoprobe^20^ and a Raman nanoprobe^21,22^ were used in fluorescence endoscopy to detect CRC. However, improvements in sensitivity and tumor specificity that yield greater signal-to-noise ratios (SNRs) would lead to better outcomes. Passive targeting of PEGylated nanoparticles that result from the enhanced permeability and retention (EPR) effect in cancer tissues can provide a fluorescent signal;^21,22^ however, this could be improved with the enhanced retention offered by binding and internalization of targeted molecular probes. Carcinoembryonic antigen (CEA) is one of the most widely available serum markers in patients with diagnosed CRC. Expression of CEA was found to be elevated in most types of advanced CRC adenocarcinomas.^23,24^ Nanoparticles directed at this molecular target in fluorescence endoscopy could link serum and stool screening tools to the imaging and provide an integrated CRC detection regimen.

Detection of CRC using fluorescent silica nanoparticles (FSNs) can yield increased signals for noninvasive imaging^25,26^ relative to conventional fluorescent dyes, and their silica backbone, which is transparent and biodegradable, offers advantages over other types nanoparticles (*e.g.,* QDs, metal clusters, chalcogenides).^27^ FSNs are superior to silicon QDs since they do not require complex or hazardous synthetic conditions and fluorescence in the near-infrared region.^28^ The silica backbone stabilizes hydrophobic fluorescent dyes on their surfaces and can be further functionalized with targeting agents making the FSNs efficient, high quantum yield (QY) fluorescent molecular probes.^29,30^ In addition, they are simple to synthesize, nontoxic, and size-controllable and have surfaces that are easily modified to allow efficient biomolecular interactions that can lead to binding and internalization into the target cells.^31^ FSNs are biodegradable,^32^ allowing for clearance and excretion from the body, which addresses both circulation and toxicity concerns.^33−36^ They can also be modified by substituting various dyes with different wavelengths of excitation/emissions, which can lead to multiplexed fluorescence imaging.^37,38^ The reduced autofluorescence and deeper penetration of light in the near-infrared (NIR) region of the spectrum suggest that NIR-emitting FSNs would have greater sensitivity and the ability to detect invasive CRCs that are considered to have an unfavorable prognosis.^25,26^ This, coupled with the efficient surface tunability of FSNs, enables them to be tailored for optimal disease-specific targeting and enhanced imaging output.

**Scheme 1:**
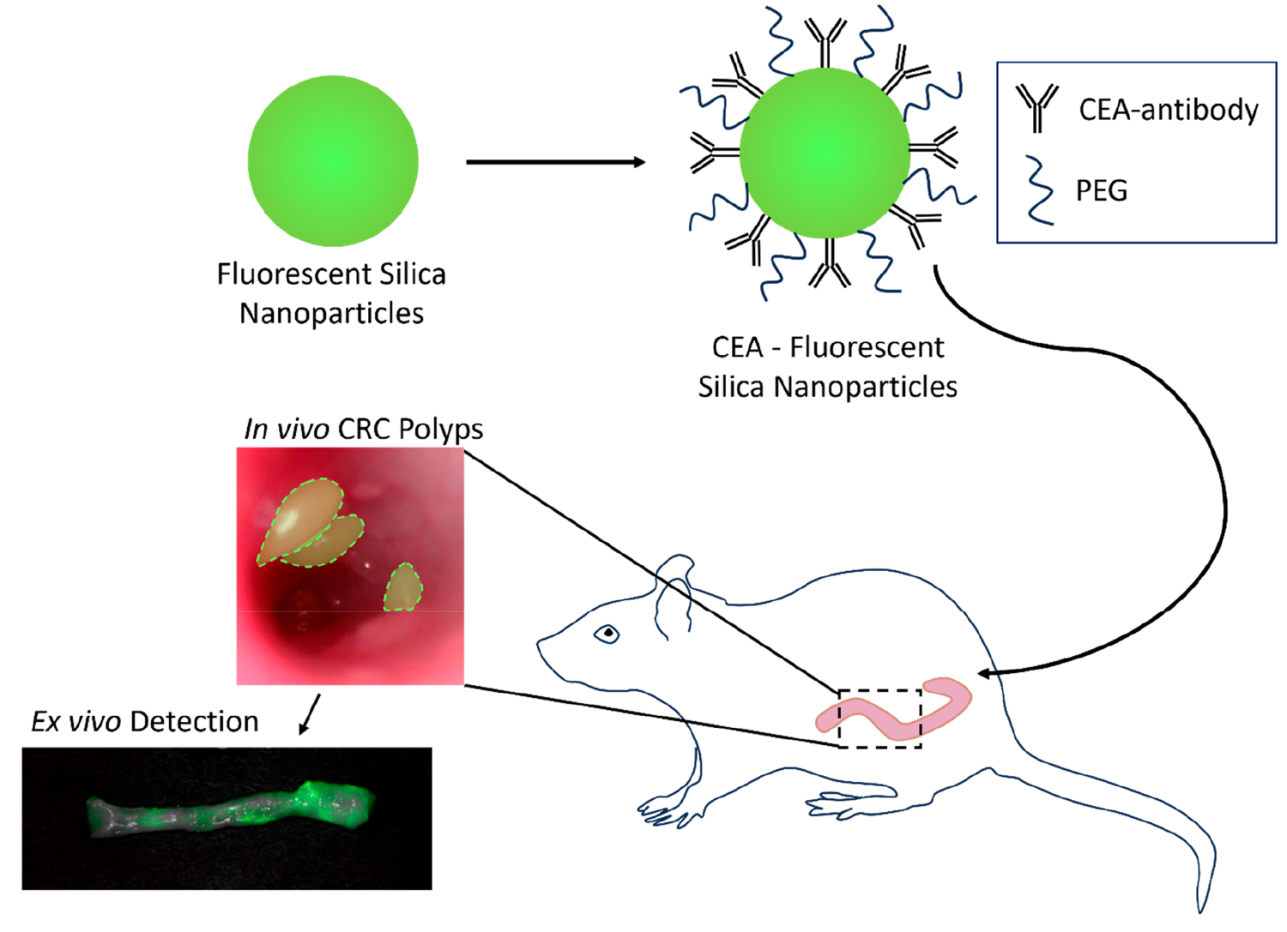
Study Design Showing *In Vivo* Targeting of CEA-FSNs in Rodent Models of CRC

This study aims to develop and test a NIR fluorescent (NIRF) nanoparticle with biodegradability that binds to overexpressed CEA on CRC enabling imaging of tumors with microendoscopes (Scheme 1). We confirmed binding and cellular uptake of the CEA antibody-conjugated FSNs (CEA-FSNs) that correlated with CEA expression on CRC cells. We performed NIRF imaging in two rodent models of CRC to reveal the potential of molecular targeting with CEA-FSNs *in vivo* imaging. In addition, *ex vivo* NIRF imaging was performed on histological sections to assess the target-specific binding of CEA-FSNs to tumor tissue that is afforded by the anti-CEA antibody.

## 2 Materials and Methods

### 2.1 Synthesis of Silica Nanoparticles

FSNs were prepared based on a modified Stöber reaction with minor modifications. Briefly, CF800-maleimide (150 μmol) was reacted with (3-mercaptopropyl) trimethoxysilane (MPTMS; 2 μmol) to yield silane-appended CF800 (CF800-MPTMS; Biotium, Fremont, CA), which was used without further purification. CF800-MPTMS was added to 2-propanol (50 mL) containing ammonium hydroxide (3 mL, 28% v/v) and tetraethyl orthosilicate (TEOS, 1 mL, 99.999% v/v). After 30 min to 2 h of stirring, FSNs were collected by centrifugation (10 min; 10 000 rpm; 25 °C) and washed once with ethanol and once with DI water. Then FSNs were exposed to tetrahydrofuran (THF) to activate thiol groups and then combined with thiolated poly(ethylene glycol) (SH-PEG-COOH; 20 mg) to form PEGylated FSNs that would allow for additional surface modifications as needed.

### 2.2 Characterization of Nanoparticles

Dynamic light scattering (DLS, Zeta Sizer Nano, Malvern Instruments) was used to evaluate the hydrodynamic diameter and zeta potential of the FSNs. The morphology of the nanoparticles was observed using a 2200FS transmission electron microscope (TEM) (JEOL, Japan). The UV absorption and fluorescent intensities of FSN nanoparticles were measured using an M5 fluoroimeter (Molecular Devices, San Jose, CA). Cellular imaging was performed with optical microscopy (Keyence fluorescence microscope, Osaka, Japan). The limit of detection of FSNs was determined by imaging a concentration series of FSNs (threefold dilution factor) on a Pearl Trilogy NIRF imaging system (LI-COR Biosciences, Lincoln, NE).

### 2.3 Biodegradation Studies

*In vitro* biodegradation studies of FSNs were performed in simulated body fluid (SBF), and analyses were via TEM. FSNs were added into SBF solution (pH 5) at a final concentration of 0.1 mg/mL FSNs. This solution was magnetically stirred slowly (100 rpm) at 37 °C for 7 days (d). A small amount of degradation solution was taken out for TEM analysis on days 1, 3, and 7.

### 2.4 Cell Culture

Two human CRC cell lines, HT29 and HCT116, and a human epithelial cell line, CCD841coN (ATCC, Manassas, VA), were used in this study. Cells were maintained in Dulbecco’s modified eagle’s medium (DMEM; Thermos Fisher, Waltham, MA) supplemented with 10% fetal bovine serum and penicillin/streptomycin (1%). These adherent cell lines were cultured in humidified atmosphere containing CO_2_ (5%) at 37 °C.

### 2.5 Western Blots

Total proteins were isolated from HT29 and HCT116 cells using radio-immunoprecipitation assay buffer (Sigma-Aldrich, St. Louis, MO). Each sample was loaded onto a 4–20% polyacrylamide gradient gel with sodium dodecyl sulfate (SDS). After electrophoresis, proteins were transferred to PVDF membranes (Bio-Rad, Hercules, CA), and membranes were blocked with skim milk (5%) for 1 h at room temperature. The membranes were incubated overnight at 4 °C with primary antibody directed at CEA (#2383S, Cell Signaling Technology, Danvers, MA; diluted 1:1000), epithelial growth factor receptor (#4267S, EGFR; Cell Signaling Technology, Danvers, MA; diluted 1:1000), vascular endothelial growth factor receptor (#ab32152, VEGFR; Abcam, Cambridge, U.K.; diluted 1:500), and β-actin (#A5451, Sigma-Aldrich, St. Louis, MO; diluted 1:5000). Membranes were incubated with horseradish peroxidase-conjugated antirabbit or antimouse IgG (Cell Signaling Technology, Danvers, MA). The signal intensity was measured using ChemiDoc (Bio-Rad, Hercules, CA).

### 2.6 Preparation and Characterization of Antibody Conjugation Nanoparticles

CEA antibodies were conjugated to PEGylated FSNs by EDC-NHS coupling reaction. Briefly, PEGylated FSNs were pelleted by centrifugation (10 min; 10 000 rpm; 25 °C) and resuspended in MES buffer (0.5 mol; pH 6). This suspension was combined with 1-ethyl-3-(3-(dimethylamino)propyl) carbodiimide (EDC) and sulfo-NHS to form NHS-esters. Then, the CEA antibody was added to the solution and stirred slowly overnight to allow for conjugation. After this, the particles were washed twice in DDI water (10 min; 10 000 rpm; 25 °C). Finally, antibody conjugation was verified by dynamic light scattering indicating a shift in size and zeta potential.

### 2.7 Cell Viability Test

We performed MTT (3-(4,5-dimethylthiazol-2-yl)-2,5-diphenyltetrazolium bromide) assay for FSNs. HT29 cells were seeded at a density of 1 × 10^4^ cells/well in a 96-well plate. The cells were then allowed to attach to the plate by overnight incubation in humidified atmosphere containing CO_2_ (5%) at 37 °C. In each well, the culture medium was replaced with fresh medium containing the samples diluted in the medium for treatment. All treatment samples were provided at concentrations ranging from 0 to 10 μg/mL and incubated for 24 h. At specific time intervals, 10 μL of MTT (5 mg/mL in phosphate-buffered saline) was added and incubated for 4 h, followed by removal of the culture medium. Subsequently, 100 μL of dimethyl sulfoxide (DMSO) was added individually to each well to dissolve. Then, absorbance was measured at a wavelength of 570 nm using a microplate reader.

### 2.8 Cellular Imaging

For NIRF imaging, cells were seeded in 6-well plates at a density of 1 × 10^5^ cells per well and allowed to adhere for 24 h in humidified atmosphere containing 5% CO_2_ at 37 °C. Cells were treated for 24 h with 10 μg/mL of nanomaterials in DMEM medium. At the end of treatment, the cells were washed with phosphate-buffered saline (PBS), and images were taken by a Pearl Trilogy NIRF imaging system (LI-COR Biosciences, Lincoln, NE). For microscopy imaging, cells were seeded in 4-chamber slides at a density of 1 × 10^4^ cells per chamber for 24 h. Cells were treated for 24 h with 10 μg/mL of nanomaterial with cell culture medium. After treatment, cells were washed 3 times with PBS, and the slides were mounted using Prolong Gold reagent with 4′,6-diamidino-2-phenylindole (DAPI). Fluorescence images were observed using a Keyence imaging system with DAPI and Cy7 filters.

### 2.9 Animal Models and *In Vivo* Imaging

All procedures involving animal studies were approved by the Institutional Animal Care and Use Committee (IACUC) of Michigan State University, while animal care and wellbeing throughout the study were monitored by the Center for Animal Resources (CAR) at Michigan State University. For tumor xenograft mice, HT29 cells (1 × 10^6^ cells) were implanted subcutaneously in the left thighs and HCT116 (1 × 10^6^ cells) cells were implanted subcutaneously in the right thighs of 8-week-old male BALB/c nu/nu mice (Jackson Laboratory, Bar Harbor, ME). The mice were used for NIRF imaging studies when the tumor volume reached 100–300 mm^3^. CF800-labeled CEA-FSNs and PEG-FSNs were intravenously injected (10 mg/kg) in tumor-bearing mice (*N* = 3 for each group), and NIR images were taken by a Pearl Trilogy NIRF imaging system at various time points up to 48 h after injection. The mice were kept under anesthesia with 2% isoflurane, and imaging was performed with white-lighted filter and 800 nm filter. The regions of interest (ROI) were drawn and calculated by ImageJ software.

Polyposis in the rat colon (PIRC) rats with an APC gene mutation that spontaneously develop intestinal polyps were obtained from the rat resource and research center (RRRC, Columbia, MO). Six-month-old male PIRC rats (*N* = 3) received CEA-FSNs (1 mg/kg) via topical application using a tube attached next to the commercial hand-held endoscope (DEPSTECH, London, U.K.), and at the same time, white-light endoscopy images were captured using the DEPSTECH program. After 10 min of incubation, PBS was applied through the tube to wash away excess particles, and the intestines were excised to obtain NIR fluorescence images by a Pearl Trilogy NIRF imaging system.

### 2.10 Immunofluorescence Staining in Intestine Tissues

PIRC rat intestines were excised and prepared for frozen embedding. Sections (4 μm) from the center of the tissue were used for staining. For immunofluorescence staining, the tissue was permeabilized in PBS Triton X-100 (5%) for 5 min and blocked for 1 h at room temperature with normal goat serum diluted 1:30 in PBS. The primary antibodies were treated overnight at 4 °C using anti-β-catenin antibody (#8480S, Cell Signaling Technology, Danvers, MA; diluted 1:1000) and anti-CEA antibody (#2383S, Cell Signaling Technology, Danvers, MA; diluted 1:1000). After washing with PBS, Alexa 488 (#A-11094, Invitrogen, Grand Island, NY; diluted 1:300) and Alexa 555 conjugated secondary antibodies (#A-21422, Invitrogen, Grand Island, NY; diluted 1:300) were added to the tissues which were then incubated for 2 h at room temperature. After staining, the slides were mounted using Prolong Gold reagent with DAPI (#P36931, Invitrogen, Grand Island, NY). Fluorescence images were observed using a THUNDER microscopy system.

### 2.11 Statistical Analysis

Statistical analysis was performed in GraphPad. All data are shown as means ± SDs. Statistical significances were determined using the student’s unpaired *t* test, and P-values of <0.05 were considered statistically significant.

## 3 Results

### 3.1 Synthesis and Characterization of Nanoparticles

**Figure 1:**
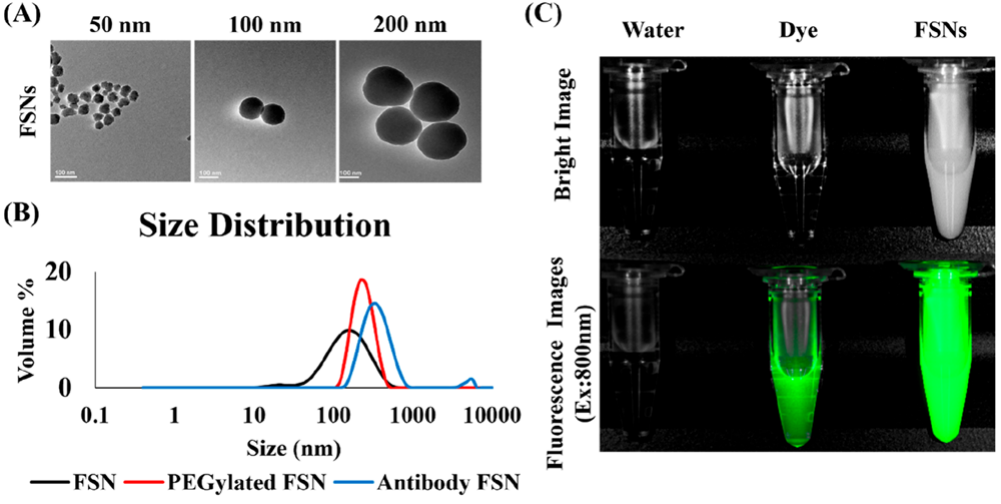
(A) Sizes of the three different FSNs were confirmed through TEM imaging (scale bar = 100 nm) to be 50, 100, 200 nm, and (B) size increase of FSNs (128 nm) after PEGylation (224 nm) and CEA antibody conjugation (341 nm) was confirmed through hydrodynamic diameter measurement by DLS. (C) FSNs (50 nm) showed 2.2 times stronger NIRF than fluorescence dye (CF800) as determined by imaging (Pearl imaging system, LI-COR).

FSNs were prepared using a modified Stöber reaction with silica-based MPTMS covalently linked with CF800, followed by surface modification with PEG by thiol–thiol interaction (Figure S1). Covalent dye linkage was chosen over encapsulation to prevent dye leaching and degradation of signal intensity over time.^39^ The zeta potential of the FSNs was +0.617 mV. In the synthesis, the reaction time mainly controlled the particle size. As shown in Figure 1A, after 30 min of stirring, the size was around 50 nm; at 1 h, the size was around 100 nm; and at 2 h the size was around 200 nm, which was confirmed by TEM. By DLS measurement, the hydrodynamic diameter for the 50 nm FSNs was found to be 128 and 224 nm before and after PEGylations indicating successful conjugation of PEG on the particle surface (Figure 1B). Differences in size as measured by TEM and DLS can be explained by solvent effects or underestimation of silica layers by TEM.^36^ An increase of hydrodynamic diameter was revealed to be 341 nm after further CEA antibody conjugation on the FSNs. NIRF images revealed dye conjugation; the resulting NIRF from the particles was confirmed, and FSNs were shown to be 2.2 times higher as fluorescent than a dye-only control (Figure 1C), due to the confinement of dye molecules on nanoscale silica surfaces.^30^

### 3.2 Biodegradation of Nanoparticles

**Figure 2:**
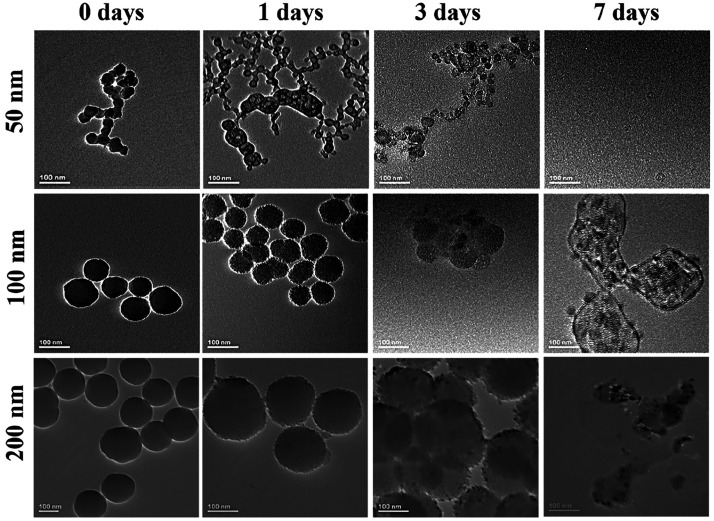
Biodegradability of FSNs confirmed by TEM imaging. The different-sized FSNs were checked for their morphology at different time points (0, 1, 3, 7 days) in SBF solution, and small FSNs (50 nm) were confirmed to be completely degraded by day 7, while larger particles (100, 200 nm) remained intact after 7 days (scale bar = 100 nm).

We have investigated the degradation profile of the particles in biological media by observing particle size variation over time. We have observed that smaller particles (50 nm) degrade faster than bigger particles (100 and 200 nm). We incubated different sizes of FSNs in SBF at 37 °C and observed that FSNs degraded as indicated by the change in color from dark brown to light brown. We noticed that while 50 nm FSNs deteriorated after 24 h, 100 and 200 nm FSNs did not start degrading until 72 h based on TEM images (Figure 2). Furthermore, these 50 nm-sized FSNs exhibited no toxicity when treated with cells, showing over 85% survival rate even at 10 μg/mL concentrations (Figure S2). With this, we have shown that we created bright, biodegradable, biocompatible, and simple-to-synthesize FSNs ideally suited to detecting CRC.

### 3.3 Antibody Selection

**Figure 3:**
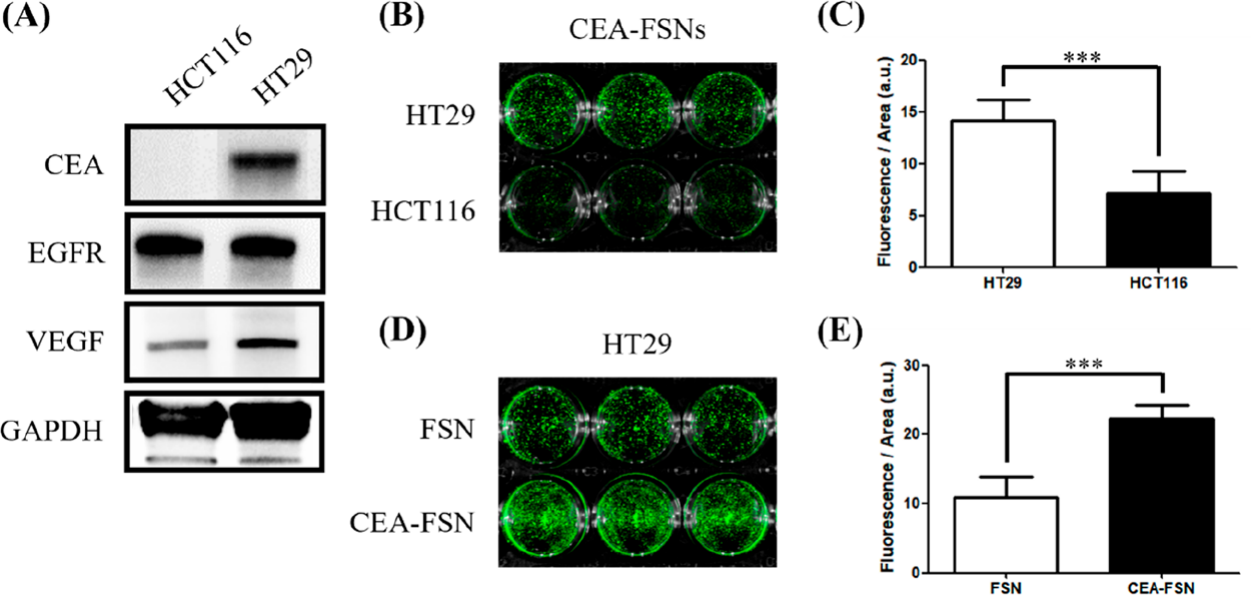
(A) Different levels of protein expression for CEA, EGFR, and VEGF were confirmed in two different CRC cell lines (HT29, HCT116) supporting the use of the CEA antibodies for CRC targeting. (B, C) CEA-FSNs were confirmed to bind more in HT29 than in HCT116. (D, E) Increased uptake of CEA-FSNs was confirmed compared to the nontargeted FSNs in HT29 cells (*N* = 3, error bars = SD, p-value: ***p < 0.0005).

Western blot analyses revealed that HT29 cells exhibited high expression of EGFR, VEGF, and CEA; HCT116 cells had lower expression of VEGF compared to HT29 cells and showed no expression of CEA (Figure 3A). In the normal colon epithelial cell line (CCD841coN), there was no protein expression of VEGF and CEA but high expression of EGFR (Figure S3). Based on these Western blot data, we strategically selected CEA as the most promising CRC biomarker for targeted nanoparticle delivery. This decision was influenced by the significant differential expression of CEA in CRC cell lines, where there was high expression in HT29 and absence in the HCT116 cells and normal colon cells (CCD841coN cells).

### 3.4 *In Vitro* Imaging of Targeted FSNs

**Figure 4:**
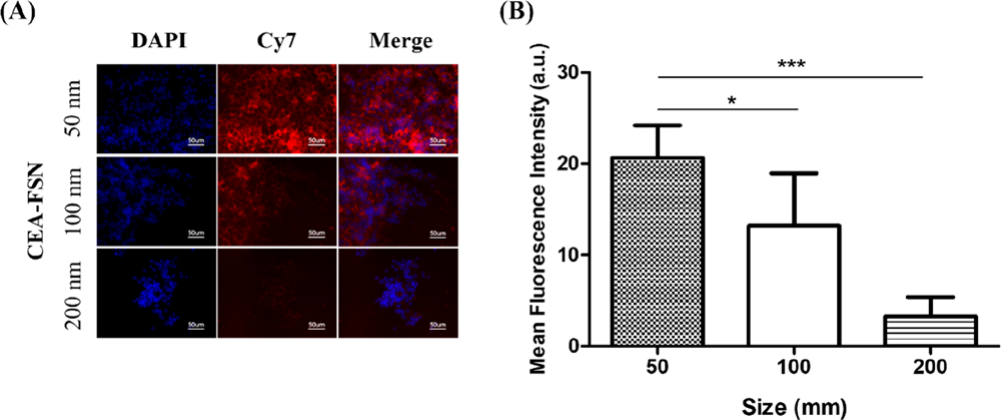
Cellular uptake of different-sized (50, 100, 200 nm) CEA-FSNs was confirmed. After treatment of CEA-FSNs in CEA-expressing HT29 cells for 24 h, the uptakes of CEA-FSNs were imaged by Cy7 channel using a fluorescence microscope (A) (scale bar = 50 μm), and the mean fluorescence intensity was measured from the images (B). (*N* = 5, error bars = SD, p-value: *p < 0.05, ***p < 0.0005).

We validated CEA-FSNs for their specificity and targeting efficiency using CEA-positive CRC cells. After incubating the cells for 24 h, CEA-FSNs displayed more binding to HT29 cells (CEA positive) compared to HCT116 cells (CEA negative) (Figure 3B, Figure S4). In the NIRF images, HT29 cells exhibited approximately twice the uptake of CEA-FSNs compared to HCT116 cells, with statistical significance (*p* = 0.005) (Figure 3C). Moreover, the CEA-FSNs revealed substantially enhanced uptake by target binding in HT29 cells in comparison to nonspecific binding with FSNs (Figure 3D and Figure S4), where the uptake was approximately 2.2 times higher in HT29 cells (*p* < 0.001) (Figure 3E). Fluorescence microscopy provided supporting evidence for the uptake of CEA-FSNs into CEA-positive cells. Using the Cy7 filter, which allows visualization of the NIRF signal from the particles, the microscopic images indicated that the smaller the size of the FSNs, the more pronounced the uptake in HT29 cells (Figure 4A and Figure S4). Quantification of fluorescence intensity showed statistically significant differences in uptake in HT29 cells for each FSN size, with smaller FSNs (50 nm) exhibiting stronger fluorescent signals (Figure 4B) despite not having higher fluorescence quantum yield than the other sizes. The quantum yields of 50, 100, and 200 nm particles were comparable and were found to be equivalent to the quantum yield of the dye (Φ = ∼0.1). These results demonstrate that CEA-FSNs efficiently bind to CEA-positive cells, highlighting their potential as a promising nanomaterial for targeted delivery in CRC therapy.

### 3.5 *In Vivo* Imaging of Targeted FSNs

**Figure 5:**
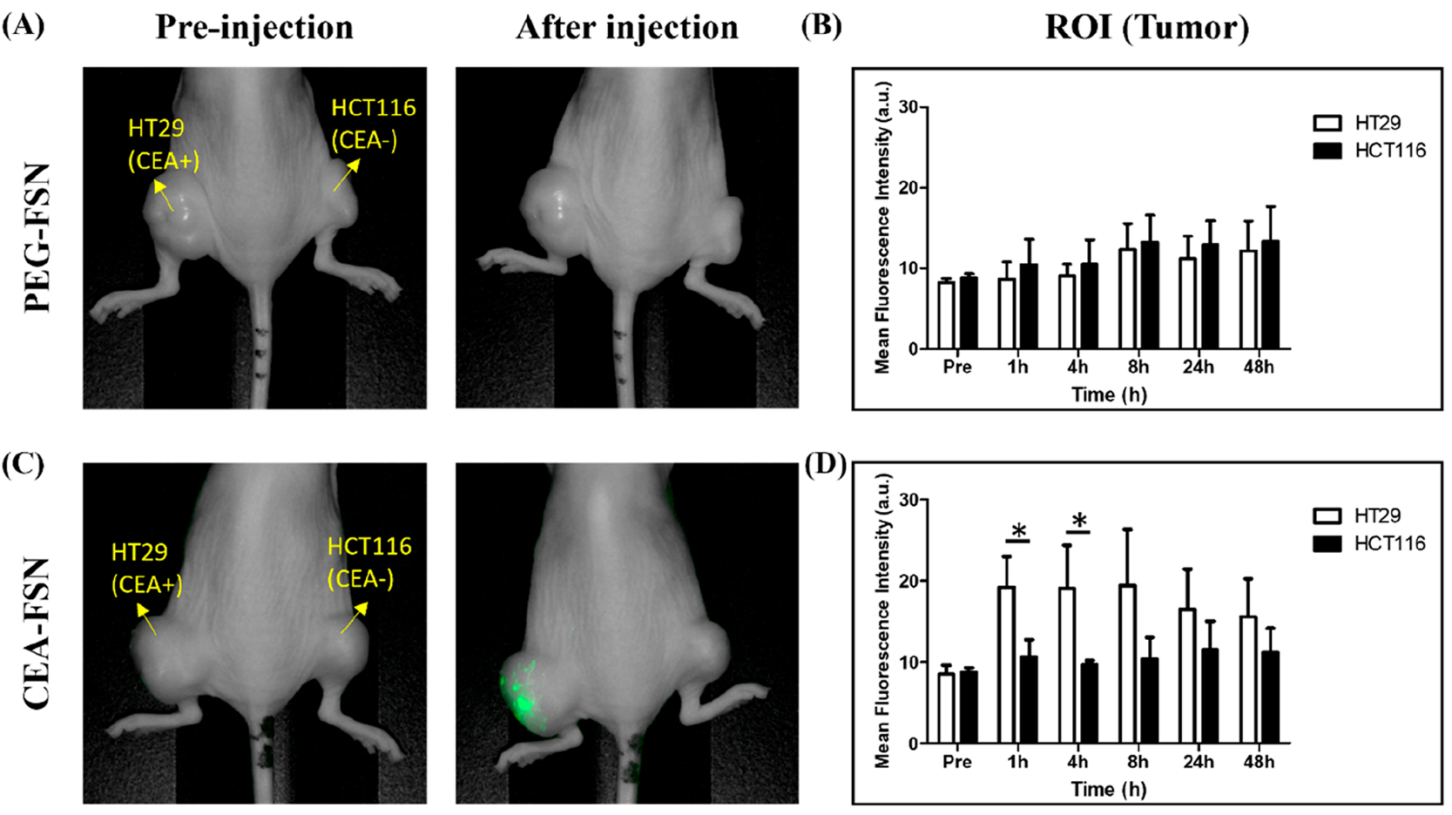
Targeted accumulation of PEG-FSNs and CEA-FSNs (i.v. injection) in CRC xenografts in mice was confirmed by a NIRF imaging system. Cell line and CEA expression indicated in yellow with yellow arrows. (A, B) There was no difference in the tumor accumulation of PEG-FSNs between HT29 and HCT116, but (C, D) the CEA-FSNs accumulated more in HT29 tumors than in HCT116 tumors at 1 and 4 h after injection. (*N* = 3, error bars = SD, p-value: *p < 0.05).

Next, we validated the ability of CEA-FSNs to target tumor xenograft mice implanted with CRC cells (HT29, HCT116) after intravenous (i.v.) injection. We performed NIRF imaging at various time points (0, 1, 4, 8, 24, 48 h) after i.v. particle injection (Figure S5). Untargeted PEG-FSNs exhibited no significant difference in uptake within the tumor region, regardless of CEA expression in the cells (Figure 5A, B). However, CEA-FSNs showed an early increase in uptake at 1 and 4 h after injection, specifically in CEA-positive HT29 tumors (Figure 5C). A twofold difference in fluorescence intensity was confirmed in the ROI of the tumors comparing CEA-positive HT29 to negative HCT116 at both 1 and 4 h after injection (Figure 5D). Fluorescence imaging of the systematic targeting was made possible using CF800 NIR fluorescent dye in the CF800-FSN as compared with dyes in the visible spectrum, like FITC, which are difficult to image in biological systems due to the background from the tissue. These results indicated that targeting CRC biomarkers promoted binding and active uptake of nanoparticles by the tumor cells, resulting in enhanced systemic targeting of CEA-FSNs compared to passive accumulation of PEG-FSNs. Additionally, 50 nm-sized nanoparticles revealed early uptake by the tumor but did not remain in the tumor region for an extended period; decreases in signal intensity were observed at 24 and 48 h.

### 3.6 Detection of CRC Polyps

**Figure 6:**
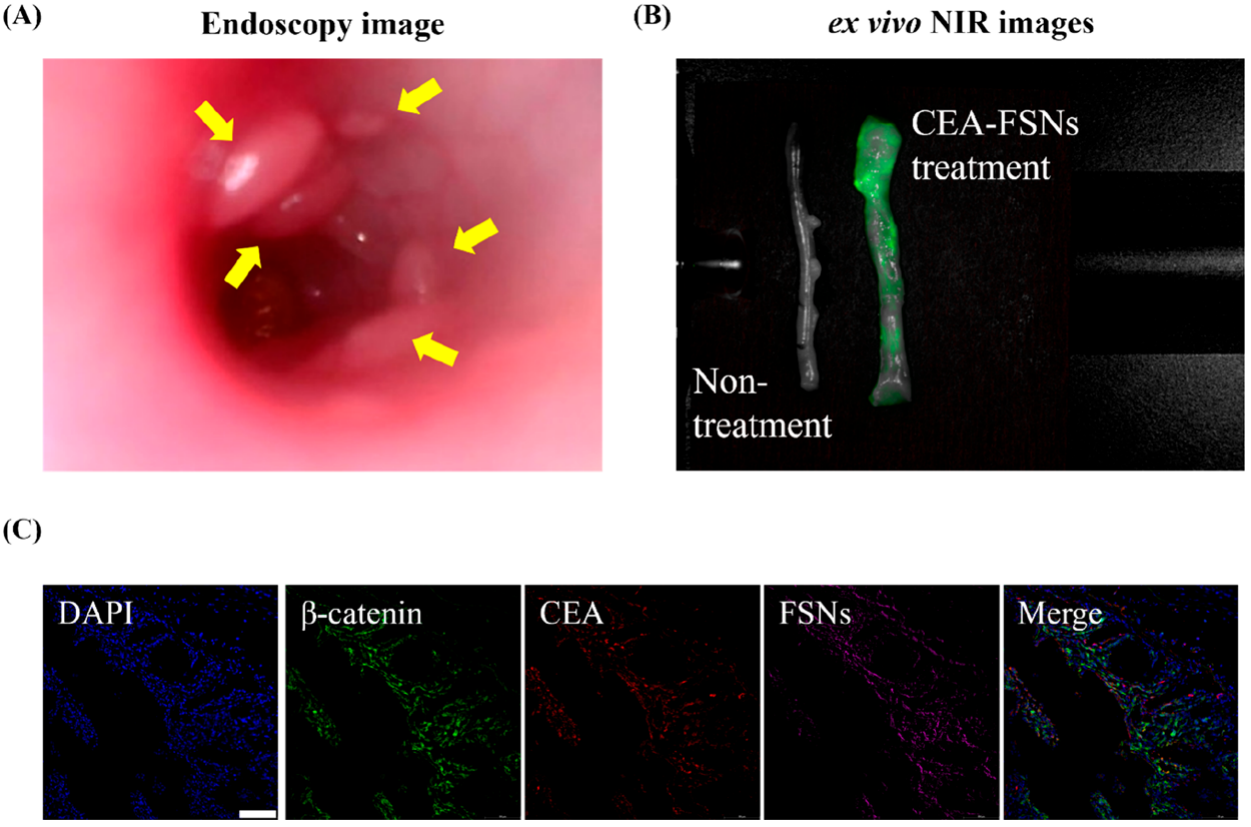
Target specific accumulation of CEA-FSNs in PIRC rats was confirmed by macroscopic and microscopic imaging of excised tissues. (A) Ab-FSNs were topically applied to polyps (yellow arrows) that were observed by white-light endoscopy in the rat colon. (B) After washing twice with PBS, a NIRF signal was confirmed in the excised intestine tissue. (C) The targeting binding of CEA-FSNs to the polyps was cross-validated in tissue sections with immunofluorescence imaging. The NIRF signals from CEA-FSNs (purple) are well overlaid with the signals from a CRC marker (β-catenin, green) and CEA (red) (scale bar = 100 μm).

The cell culture experiments and use of xenografts in mice supported the study of FSN binding in the spontaneous tumor model, PIRC rats, where naturally occurring polyps were observed by colonoscopy following topical application of nanoparticles to the intestinal surface. Transmucosal drug delivery after topical administration of silica nanoparticles is possible due to their small size and hydrophilic exterior.^40,41^ Using white light, polyps in the intestine were successfully detected with endoscopy (Figure 6A), and subsequently, after topically applying CEA-FSNs, the strong NIRF signals were observed in the excised intestinal tissue (Figure 6B). Using immunofluorescence imaging on excised tissue sections, the signals emitted by CEA-FSNs colocalized with the signals from CRC markers (β-catenin) and CEA antibodies (Figure 6C). This colocalization of signals validated the specificity of CEA-FSNs. This indicates that CEA-FSNs specifically target CEA-expressing tissue and do not exhibit nonspecific binding to healthy tissues making them the ideal particle to use for this application especially when compared to nonantibody-conjugated FSNs.^21^ The *ex vivo* imaging results support the use of CEA-FSNs as a reliable and precise molecular imaging agent, diagnostic, and targeted therapy for CRC.

## 4 Discussion

In this study, we evaluated the binding and uptake of biodegradable FSNs conjugated with CEA antibodies, compared to untargeted PEGylated FSNs in colon cancer cell lines and tumor tissues of CRC animal models. CEA was used as a target-specific biomarker for directed binding and uptake in CRC. Using NIRF imaging of cell lines with different CEA expression levels, we confirmed that CEA-FSNs track CEA and also bind to tumors *in vivo* after systemic injection into murine xenograft models. Additionally, we could identify the NP-enriched CRC tissues present in the intestine with *ex vivo* imaging following topical application of CEA-FSNs on the intestinal surface of PIRC rats.

We used the HT29 and HCT116 colon carcinoma cell lines, which are well-established and widely used in CRC research. Our primary aim was to confirm a suitable biomarker for target-specific imaging of CRC tissues with FSNs. For initial marker selection, we studied the expression levels of three prominent biomarkers: VEGF, EGFR, and CEA; VEGF and EGFR are being utilized for the treatment of CRC in patients using the antibodies bevacizumab (targeting VEGF-A),^42^ aflibercept (targeting VEGF-A and VEGF-B),^43^ panitumumab, and cetuximab (targeting EGFR).^44^ CEA is a commonly used serum biomarker with high specificity for diagnosing CRC. Elevated CEA levels have been observed in the blood samples of CRC patients, with overexpression detected in 90–95% of CRC cases.^45−47^ Tiernan et al. revealed that CEA displayed the most significant differential expression between tumor and normal tissues.^48^ Moreover, in this study, CEA demonstrated significant differential expression between positive and negative lymph nodes, outperforming other CRC markers such as TAG-22, FRα, and EGFR.^48^

The FSNs of different sizes (50–200 nm) were successfully synthesized using different durations of a simple reaction. All FSNs were found to be uniformly distributed and monodisperse. Small nanoparticles are known to penetrate into tumor tissue and to be excreted quickly.^49,50^ Our *in vitro* cell uptake data indicated that the 50 nm CEA-FSNs bound to and were taken up into target cells significantly better than the 100 or 200 nm particles. The biodegradability profile has a significant impact on the potential clinical application of nanoparticles.^51^ The biodegradation rates depend on particle dimensions (*e.g.,* size and shape). We found the small-sized FSNs (50 nm) were also more readily degraded compared to the larger particles (100 or 200 nm) in a biological medium. For enhanced tissue enrichment and biodegradability, the results indicate that the 50 nm FSNs were well-suited for conjugation to the selected antibody targeting CEA. In the translation of the 50 nm particles to large animal models and humans, if we find that they are excreted too quickly, we can combine them with a larger, biocompatible carrier like albumin to enhance their circulation time and tumor retention, while preserving their ability to infiltrate tumors.^52−54^

The formulation of the FSNs evaluated in this study could accommodate other dyes to enable multiplex fluorescence imaging, although here we only used CF800. The FSNs could be conjugated with other dyes with nonoverlapping wavelengths of excitation/emission. These FSNs could then be conjugated to other candidate antibodies, allowing them to target other cancer markers. Multiplexed imaging with a cocktail of FSNs could increase the diagnostic specificity and sensitivity and would enable ratiometric imaging to refine the output.^37,38^ In the case of CRC, the use of specific antibodies could be used to distinguish between common adenocarcinomas and uncommon cancers like sarcomas. Additionally, groups of antibodies could be selected to target different stages of the same cancer allowing for more accurate staging and treatment planning. Finally, antibodies could be selected to tag multiple immune cells associated with disease prognosis allowing us to determine more personalized diagnostic and treatment schemes.^55^

The NIR-I region of the spectrum (650–950 nm) has been identified as the first biological window, characterized by reduced absorption by blood and water and decreased scattering by biological structures, *e.g.*, mitochondria, within cells and organs.^56^ Despite these advantages, NIR-I fluorescence bioimaging is still subject to tissue autofluorescence and photon scattering, limiting tissue penetration depth.^57^ Recent experimental and simulation studies have revealed that we can achieve a higher SNR by utilizing the second region of the NIR region (NIR-II, 1000–1700 nm), also known as the second biological window.^58,59^ The NIR-II light enables detection of signals from deeper in the tissue, in the range of centimeters, while maintaining micron-level resolution at millimeter depths, surpassing the performance of NIR-I fluorescence imaging.^60^ In CRC, histological criteria include deep submucosal invasion ≥1000 μm or deep submucosal invasion;^61^ real-time diagnosis using deep penetration is an essential adjunct to screening for CRC. Consequently, NIR-II bioimaging would improve fluorescence endoscopy by revealing physiological and pathological phenomena in CRC; however, the number of dyes in this region of the spectrum is, at present, limited.

White-light colonoscopy has been a valuable tool for visualizing the surface topology of the colorectal mucosa. However, these anatomic changes are insufficient for rapid and accurate diagnosis. To overcome this limitation, multimodal imaging systems that integrate NIRF, Raman signals, and other real-time optical detection tools with white-light endoscopy have the potential to provide a wealth of information for detection and staging. Surface-enhanced resonance Raman scattering (SERRS) nanoparticles have extremely high target-imaging specificity, are readily multiplexed with a single excitation wavelength, and have demonstrated reliable detection of precancerous lesions in animal models.^20^ Nevertheless, complicated image reconstruction processes are limiting factors. Fluorescence colonoscopy offers real-time, wide field-of-view imaging with excellent sensitivity, facilitating the screening of large surfaces, although with more limited multiplexing capability than Raman imaging. Therefore, imaging with NIRF particles can achieve the desired goal of rapidly identifying suspicious lesions in the gastrointestinal tract.^62^ Integrating NIRF imaging capabilities into white-light endoscopes will provide both the essential morphological information and red flag detection of molecular markers to further enhance diagnostic capabilities.^63^ A multimodal approach, with combined NIRF, Raman scattering nanoparticles, and real-time colonoscopy imaging, holds promise for overcoming current limitations and advancing colorectal lesion detection, ultimately leading to improved patient outcomes and more efficient medical decision-making.

## 5 Conclusions

In this study, we evaluated FSNs as a targeted imaging probe for CRC diagnosis. First, we synthesized CF800-labeled FSNs and found stable NIR signals with excellent intensity from the particles. We also demonstrated biodegradability of the FSNs, ensuring their safe and efficient utilization in future biomedical applications. By conjugating selective biomarkers of CEA antibodies to the FSNs, we verified CRC targeting as evidenced by specific binding to CEA-positive CRC cells. We confirmed that smaller CEA-FSNs have better cellular uptake and biodegradability than larger CEA-FSNs. Intravenous injection or topical application of CEA-FSNs in rodent models confirmed targeting of CEA-FSNs to tumor tissues. These promising results indicate that CEA-FSNs could be used as a surrogate imaging probe for the early diagnosis and surgical resection of the CRC. Further evaluation of the CEA-FSNs on detection sensitivity for the colorectal polyps during the colonoscopy procedure will warrant the wide availability of CEA-FSNs in molecular imaging of CRC and accelerate its clinical translations.

## Supporting Information Available

Schematic figure for nanoparticle preparation; cell viability test of CRC cell lines (HT29 and HCT116) with PEG-FSNs; western blot data information on the expression of candidate proteins EGFR, VEGF, and CEA in colon epithelial cell lines (CCD841coN cells) and CRC cell lines (HT29 and HCT116); cell fluorescence images of the uptakes results of PEG-FSNs and CEA-FSNs in HT29 and HCT116 cells; whole body images of the uptake results of PEG-FSNs and CEA-FSNs in HT29 and HCT116 xenograft mice (PDF)
