# Supplementary material for: Targeted Biodegradable Near-Infrared Fluorescent Nanoparticles for Colorectal Cancer Imaging
Source: ACS Appl Bio Mater. 2024 Apr 4;7(12):7861–70. doi: 10.1021/acsabm.4c00072 (PMC11653400; doi:10.1021/acsabm.4c00072)

Supporting information

Targeted Biodegradable Near-Infrared Fluorescent Nanoparticles for Colorectal Cancer Imaging

Seock-Jin Chung[1+], Kay Hadrick[1+], Md Nafiujjaman[1+], Ehsanul Hoque Apu[1,#], Meghan Hill[1], Md Nurunnabi[2], Christopher H Contag[1,3], and Taeho Kim[1]*

[1]*Department of Biomedical Engineering, Institute for Quantitative Health Science and Engineering, Michigan State University, East Lansing, MI 48824, USA*

[2]*Department of Pharmaceutical Sciences, School of Pharmacy, University of Texas at El Paso, TX 79902, USA*

[3]*Department of Microbiology and Molecular Genetics, Michigan State University, East Lansing, MI 48824, USA*

**Corresponding Author Email:* kimtae47@msu.edu

[†]S.C., K.H., and M.N. contributed equally to this work.

[#]*Present Address: Biomedical Sciences, Lincoln Memorial University, Knoxville, TN 37917, USA*

Figure S1. Schematic presentation of synthesis of Fluorescent Silica Nanoparticles (FSNs).

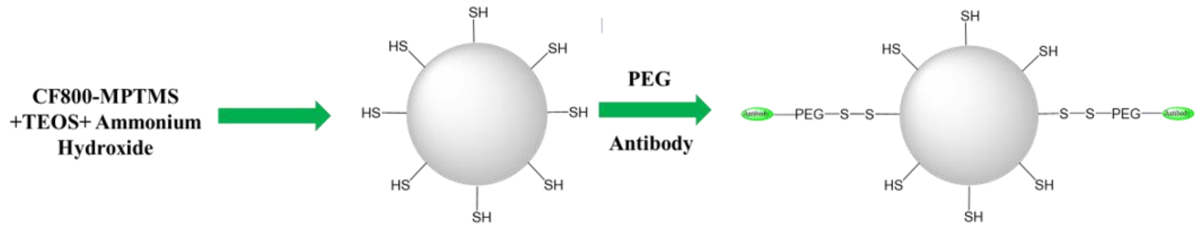

Figure S2. Cell viability of (A) HCT116 and (B) HT29 cells treated with PEG-FSNs was measured using MTT assay (N=5, error bars=SD, 5% Triton-x100).

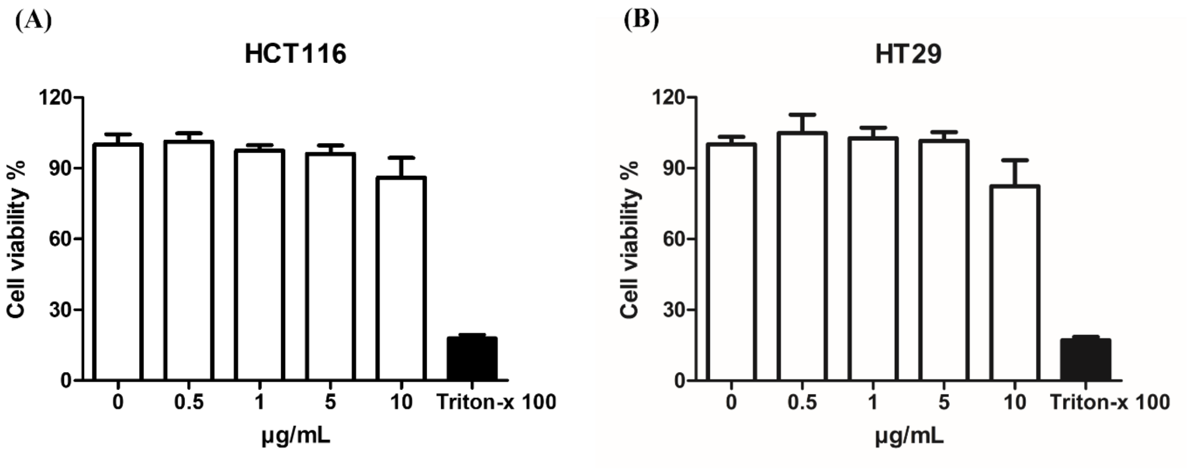

Figure S3. The expression levels of EGFR, VEGF, and CEA were confirmed through western blot in colon epithelial cell line (CCD841coN) and two CRC cell lines (HT29, HCT116).

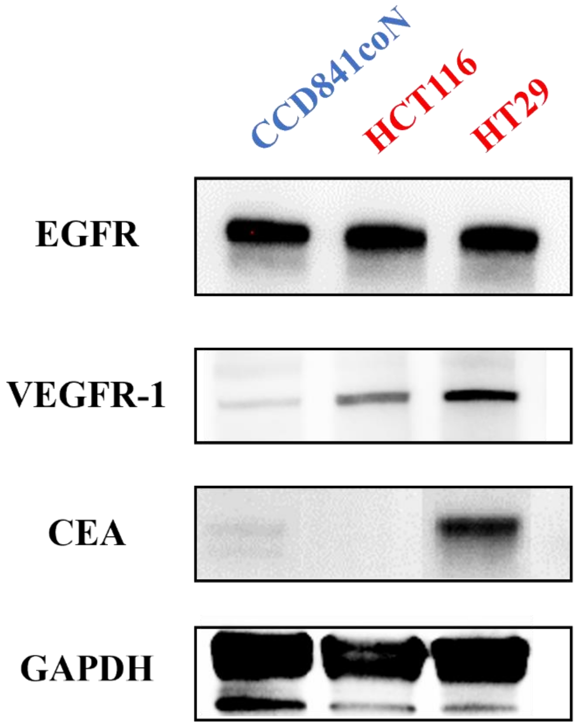

Figure S4. Cellular uptake of FSNs of various sizes (50 nm, 100 nm, 200 nm) was confirmed in high-magnification images. After treatment of CEA-FSNs or PEG-FSNs in HT29 or HCT116 cells for 24 hours, the uptake of FSNs was captured using a fluorescence microscope with the DAPI (Blue) and Cy7 channels (Red) (Scale bar=50 μm).

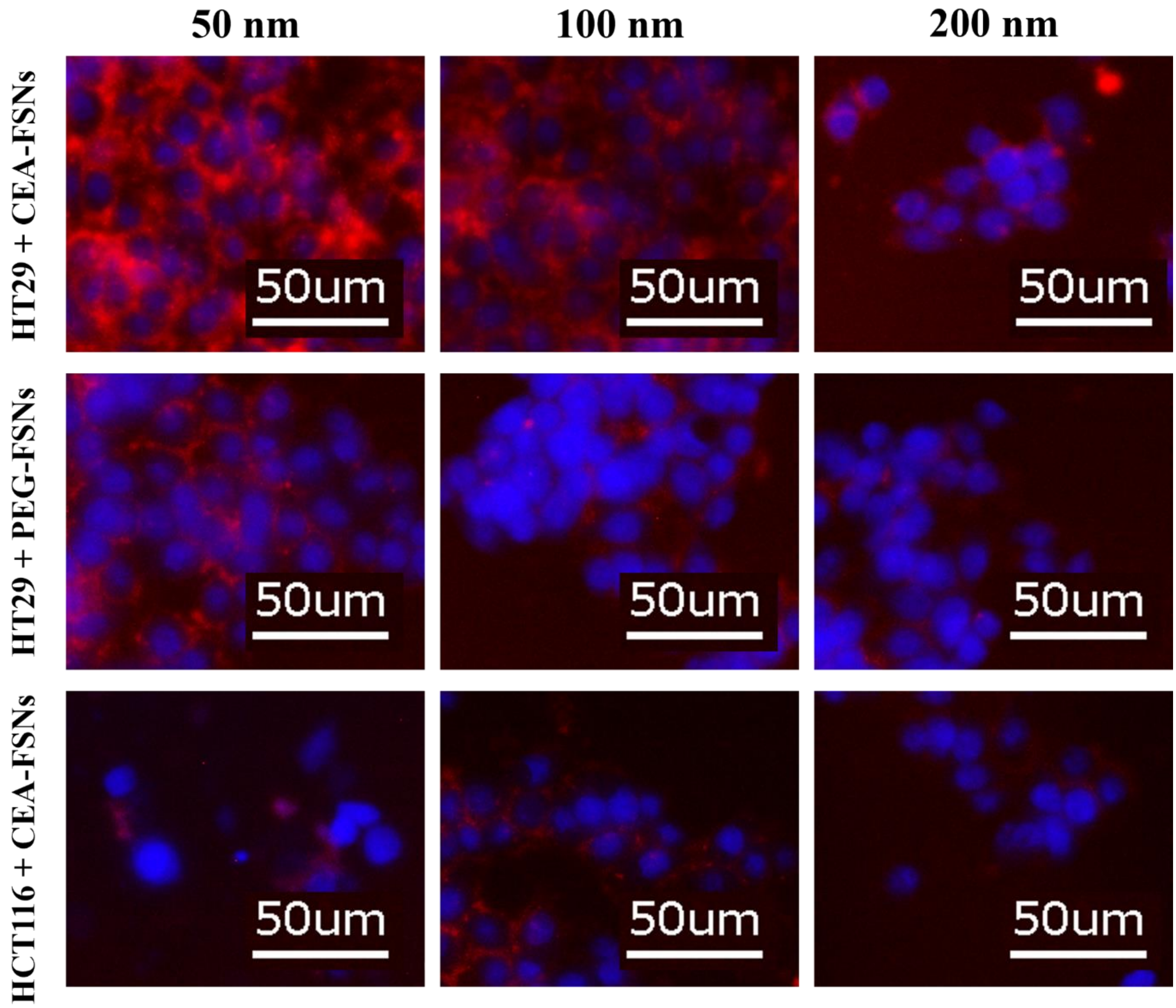

Figure S5. Targeted accumulation of PEG-FSNs and CEA-FSNs (i.v. injection) at different time points in CRC xenograft mice (whole body) was confirmed by the Pearl imaging system.

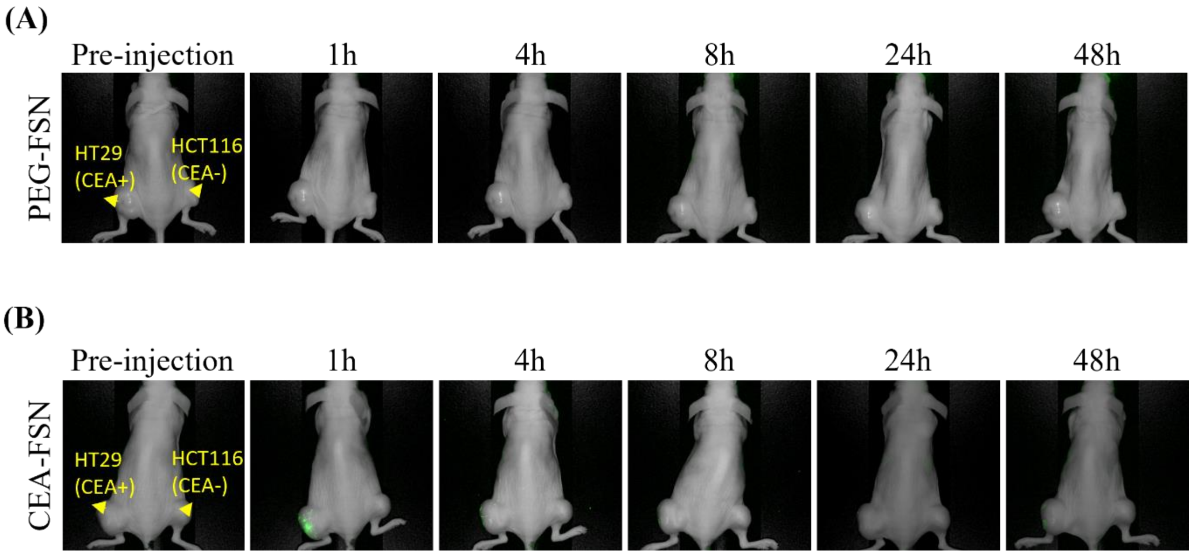

Supplement: Supplementary file 1 — mt4c00072_si_001.pdf [file mt4c00072_si_001.pdf]
